# Editorial: Mobilities, migration, and digital humanities

**DOI:** 10.3389/fsoc.2025.1715327

**Published:** 2025-10-31

**Authors:** Mayurakshi Chaudhuri, Chiranjoy Chattopadhyay, Nirmala Menon, Paul Longley Arthur, Sarah Kenderdine, Mattius Rischard, T. Shanmugapriya

**Affiliations:** ^1^Department of Social Sciences, FLAME University, Pune, India; ^2^Department of Computing and Data Sciences, FLAME University, Pune, India; ^3^Indian Institute of Technology Indore, Indore, India; ^4^Edith Cowan University, Joondalup, WA, Australia; ^5^Ecole Polytechnique Federale de Lausanne, Lausanne, Switzerland; ^6^Montana State University-Northern, Havre, MT, United States; ^7^Indian Institute of Technology (Indian School of Mines) Dhanbad, Dhanbad, India

**Keywords:** migration, mobilities, digital humanities, sociology, social networks, data visualization

The history of migration in sociological scholarship has been a history of mobilities and immobilities. This history also carries resonances and nuances reflecting global historical trajectories such as those deriving from prolonged periods of imperialism and colonialism, whose legacies are central to mobility narratives, memoirs, and archives. Such global historical trajectories often impact locally and inflect mobile people's experiences of structure, agency, and identity over generations.

In this history of migration studies, mobility as an overarching concept has been a constant that has allowed for investigation of a variety of movements of people in connection to material, ideal, and virtual flows (for details, see “The New Mobilities Paradigm” by Sheller and Urry, 2006). This connection, either between agents (humans), or between agents and networks (such as human and technology), is not new (Latour, 1996) and is often encapsulated in migration language as “social networks.” Social networks often refer to and are contingent on people's social locations (Pessar and Mahler, 2003). In other words, people's social position within interconnected power hierarchies characterizes their relations across the geographic scales, from the intimate to the transnational and global. Such interconnected networks of power can be shaped by multiple stratifying factors such as history, politics, economics, geography, and family and kinship relations. Social locations are often acquired through life circumstances and opportunities. This gives social locations their dynamism; they are fluid, changeable and multiple. This can help to socially locate people by, for example, gender, social class, race, sexuality, ethnicity, nationality, and age, and their intersections, across multiple categories.

Social networks are also characterized by people's *agency*, understood in terms of cultural geographer Massey's (1994) concept of “power geometry.” Massey argues that the interplay of time and space impacts people's geographic and social location, and therefore, in distinct ways, their relations to flows, movements, and interconnections. This does not necessarily involve people's physical movements but relates more to their networking and interconnectedness and levels of access to resources and, therefore, power. Such exercising of agency is not limited to people who physically migrate, but encompasses non-migrants too. Social networks provide information not only about migration movements, but also about the structure and dynamics of the complex mobility systems within which migrations occur (such as geo-social terrains with prolonged legacies of colonial rule exhibiting and negotiating social “pressure points” and movements across multiple geographic and social scales) (Chaudhuri and Thimm, 2018).

While recent decades have witnessed an exponential growth of migration and mobility literature with particular emphasis on social networks, the domain of migration and mobility studies has also evolved toward modes of inquiry that are more interdisciplinary. Along this trajectory, data visualization has become increasingly crucial for comprehending migration patterns, as it transforms complex data into accessible and engaging descriptions. This has enabled the domain of migration and mobility studies to cross disciplinary “boundary walls” in significant ways to work with another emerging field of interdisciplinary inquiry: Digital Humanities (DH). The field of DH has opened new analytical possibilities by using computational approaches to study various domains and dynamics, including migration. Analysis of data on digital devices, platforms and in databases has become an important focus for social scientists and humanities scholars, enabling them to gain new knowledge of migration, and to be equipped with new quantitative and qualitative tools of inquiry.

The dynamic engagement of the broad field of mobilities and migration with DH is the focus of the articles in this Research Topic. This intersection provides a timely opportunity for scholars from diverse disciplines to engage in critical discussions, explore innovative methodologies, and share their research on these interconnected themes, in global contexts.

The eight articles in this Research Topic provide a variety of perspectives on the common theme of Social Networks that has always been central to Migration/Mobility Studies and that also represents an important strand in the field of DH. Within this larger framework which all eight articles address, three articles in particular further address a subtheme that is intrinsically related to DH: Data Visualization (in this case, Visualizing Social Networks). Data visualization in migration studies is not a new phenomenon. However, what has been remarkable is the progress that has been made in conveying the *multi-dimensionality* of the data contained in often large and complex matrices of origin/destination migration flows (i.e., big data), which conventional tools such as static maps and graphs (such as flow line maps) have had very limited capability to convey.

Building on this shared emphasis on social networks, and extending it through the affordances of digital methods, the eight articles demonstrate how DH analytical tools make networked mobilities visible across very different terrains. Three articles explicitly *visualize* or *map* social ties: Mukherjee and Menon computationally probe “digital migration infrastructures” in Indian return-writing, using Python text analysis and plots to surface how ICTs mediate routes home (e.g., mobile, internet, remittances) in contemporary literature; Özdemir et al. survey refugee-oriented mobile apps and show how GIS/GPS features operationalize adaptation through locative media—literally placing social support on the map; and Rischard applies network theory to a postcolonial social science texts on Haiti, diagramming subaltern “resistance capital” to reveal how power and counter-power circulate through relationships, and modeling how DH network analysis can re-read migratory/colonial encounters in cultural archives. The remaining articles extend the social-network lens beyond visualization to *infrastructures, platforms, and archives*. Parmar theorizes community digital archives (SAADA; 1947 Partition Archive) as sociotechnical networks whose crowd-sourced records and digital connectivity reconfigure belonging, access, and historiography for South Asian Diasporas. The interdisciplinary agenda of this article positions it squarely at the migration—DH nexus. Meseşan-Schmitz et al.s' mixed-methods study of Romanian Roma highlights how interpersonal ties and prior pathways steer temporary mobility and return, clarifying networked recommendation effects alongside classic push–pull factors. Tsang and Wilkinson's analysis of rural-to-urban Chinese male migrants in livestreaming foregrounds “affective labor” and guild structures in “platformized” economies, showing how digital marketplaces become new migration infrastructures and social fields of aspiration and risk. Moratilla examines how poetry as a creative strategy in the context of the COVID-19 pandemic offered important avenues to create social networks to combat collective grief, anxiety, and desire for survival. Mariano et al.'s study on digital racism and exclusion from the decolonial perspective brings out the power of intersectionality as networks through new forms of hierarchizing difference and possibilities of resistance to colonial structures.

Together, these articles' contributions show why visualizing and modeling of networks matter. Moving from nineteenth-century flow maps to contemporary computational text mining, GIS, and graph analysis not only scales to “big/thick” data, but also brings to the surface the multi-dimensionality of mobility—its infrastructures, archives, platforms, and lived affects—now central to both Migration/Mobility Studies and the Digital Humanities.

We express our gratitude to all the contributing authors for their significant work in this Research Topic, to the Frontiers Journal team who supported the vision for this Research Topic and the associated logistical processes, to the peer reviewers who provided their feedback on the manuscripts to ensure that the research articles are in alignment with the Research Topic and that the rigor and quality standards of the Journal are upheld. As co-editors and topic coordinators, we are hopeful that these examples of intersections of *Mobility, Migration, and Digital Humanities* will be complemented and expanded by future generations of research.
